# Observations on Stone in the Urinary Bladder, and on Lithotomy

**Published:** 1790

**Authors:** James Lucas

**Affiliations:** one of the Surgeons of the General Infirmary at Leeds


### V. *Observations on Stone in the urinary Bladder, and on Lithotomy. By Mr.* James Lucas, *one of the Surgeons of the General Infirmary at Leeds.*

THE sufferings of those who labour under this painful malady, the injury they sometimes experience from improper modes of treatment, and the benefit which in such cases is occasionally derived from innocent remedies, together with the importance of lithotomy, are all of them circumstances that seem to render a farther investigation of this subject not altogether unnecessary.

The stone in the urinary bladder is a disease to which persons of all ages are liable, but more especially children, and such as are advanced in life; and males in a much greater proportion than females.

It sometimes appears to be hereditary; a sedentary life may conduce to it; but it may be doubted whether a town or country residence alters its prevalence. I have known an infant, little more than a year old, attacked with it; and as children affected by it are generally of the poorer class, I am inclined to conjecture that that it may ſometimes depend upon want of proper exerciſe and cleanlineſs. Its infrequency in the middle-aged may depend upon their uſual activity, upon their attention being leſs excited if the ſymptoms are moderate, and upon advantage being ſometimes, for a while, reaped from common remedies.

In elderly people ſuch indiſpoſition often commences in the kidnies, and therefore future attacks may, with greater reaſon, be expected; yet I remember a man in whom the complaint appeared to be local, although he had no ſymptoms of it until he was ſixty-ſix years of age.

The ready exit of ſabulous concretions, greater regularity in diet, together with a deſire to conceal ſuch ſymptoms, may ſufficiently account for their being more rarely met with in females.

I am far from concurring with thoſe who deny that diſeaſes happen by deſcent, and am of opinion that, in ſome inſtances, the prognoſtic, if not the treatment, may be influenced by this circumſtance. Two brothers are at preſent under my care with cataracts, and their father had the ſame malady, although their occupations have been different. The cauſes of hereditary reditary complaints being occult and uncertain, tend to alleviate the miserable suspense that must otherwise have been inevitably entailed on posterity.

A change from an active to a sedentary life, together with an unaccustomed indulgence in diet, may be deemed the more immediate cause of calculous concretions. An elderly gentleman, who could without inconvenience have used severe exercise on horseback, was, by a tedious confinement, rendered unable to take an airing without passing bloody urine.

Sudden alterations in the mode of living or exercise should be studiously avoided by those who may retire from active employments. Upon a similar caution being given to an acquaintance of mine, he observed, that his own experience had already suggested its necessity, and yet he had reason to attribute many violent attacks of pain in the urinary passages to his own irresolution. This town and neighbourhood abound with inhabitants who live well, and are by their occupations confined to a sitting posture, yet stone in the bladder is not in these parts uncommonly prevalent.

Urinary calculi vary much in texture and colour; nor can the peculiar sort of stone be conjectured jectured from the age of the patient, or duration of the indifpofition. Thofe which are foft and readily broken are often of a lighter colour; but the external furface may be foft, and the internal hard; and thefe are probably the fort of ftones which chiefly admit of being affected by internal medicines. The mulberry or rough ftone is ufually of a very firm confiftence, and dark colour.

When the external rough furface of a ftone is rubbed off, and ftony portions are evacuated, (a fmooth calculus remaining) fo much eafe may be procured, that a cure may appear to have been performed by a total diffolution of the ftone; but fuch benefit cannot be admitted as certain proof of a complete folvent. Equal advantage has alfo been received without any difcharge of calculous fragments: but as in every inftance yet recorded, when the bladder has been examined after the death of thofe whofe perfect cure by folvents had been boafted of, one or more ftones have been difcovered, although no recent fymptoms had happened; it feems fair to conclude, that no remedy has yet been found out capable of diffolving a ftone during the life of the patient. It is probable that medicines may not conftantly produce their falutary effects by acting acting upon the calculus, but may induce a change in the bladder itſelf, ſo that the ſtone may not equally irritate as it had previouſly done. The durable effect of opium in ſuch caſes may not improbably be thus explained.

It is ſomewhat extraordinary that ſuch oppoſite remedies, as fixed air and lixivia, ſhould have procured an evacuation of calculous concretions, and been attended with nearly equal advantage. It is alſo fortunate, that the pernicious effects of one may, in a great meaſure, be counteracted by the other. Chemical experiments upon the urine of calculous patients, and upon urinary calculi in the ſame menſtruum, may at ſome future time tend to point out remedies for this painful affection.

Diſeaſes in the proſtate gland, in other parts, or even in the vicinity of the urinary paſſages, may ſo far reſemble in their ſymptoms thoſe produced by a ſtone in the bladder, as to render a diſtinction difficult: therefore, not only previouſly to lithotomy, but alſo to the uſe of any remedy that may injure the conſtitution, the malady ſhould be clearly aſcertained. From a miſtaken notion of this kind a courſe of lixivia, in one caſe, proved fatal.

 The

The common ſigns of a ſtone in the urinary bladder are, a painful and more frequent inclination to void urine, although little at a time is diſcharged, accompanied with a teneſmus or prolapſus ani; an itching in the glans penis; a ſenſe of weight in the perinæum, a calculus having apparently deſcended from the kidney without having been evacuated, and mucus, and ſometimes blood, being mixed with the urine.

Theſe diſtreſſing ſymptoms more frequently return by fits, but may be rendered more conſtant or violent from a ſharp pointed, rough, large, or encyſted ſtone, from ſevere exerciſe, or exceſſes in diet; as well as moderated by occaſional reſt, an abſtemious regimen, and mucilaginous diuretics. Tar water, fixed air, ſoap, opium, and particularly anodyne clyſters, have been found in ſuch caſes efficacious.

The ſpeedy, powerful, and evident effects of lixivia have appeared to entitle them to a preference; but it is much to be feared that more harm than good has accrued from their uſe. Some conſtitutions, indeed, ſeem to be robuſt enough to reſiſt the pernicious qualities of the moſt potent drugs, yet fatal ſymptoms have ſucceeded a ſudden alteration in exerciſe exercife and mode of living under a courfe of lixivia; a bruife in the foot has proved mortal; a flight wound difficult of cure; and morbid fymptoms of various kinds have enfued from a continued ufe of medicines of this fort. Thefe remedies are infufficiently adminiftered to diffolve a ftone: they feldom fail to weaken the fyftem; they for a long time, at leaft, prohibit any advantage being derived from lithotomy, and render any other complaint more violent. It is much to be lamented that medicines of fuch dangerous tendency fhould be entrufted to patients, who, notwithftanding evident injurious effects, will often recommend the fame plan to others, and conclude that a painful as well as defperate difeafe requires a defperate remedy.

If I am not mifinformed, a fimilar compofition has of late been ufed not only for the ftone in the bladder, but for biliary concretions. Such credit would not be given to public noftrums of this fort, if their mifchievous confequences were fufficiently known. Although thefe compofitions may fometimes alleviate, yet they often increafe the painful fymptoms; and when they procure immediate eafe, opium may be fufpected to be difguifed in the preparation; nor is fuch an addition objectionable. The more innocent remedies have proved equally advantageous in difcharging calculous fragments, and in removing painful fymptoms. Lixivia fhould, therefore, never be employed until other means have been fedulously, though ineffectually, tried, until lithotomy has been difapproved or rejected, and efpecially until a ftone has been clearly difcovered. When, however, thefe hazardous remedies are made choice of, fuch means as have been experienced to counteract their pernicious confequences fhould be carefully intermixed. Unexpected relief has often been obtained from fimple remedies, and their peculiar advantages are, that they neither prove detrimental to the habit, nor hinder the fuccefs of an operation; and feldom fail to appeafe the moft painful fymptoms.

When opium is liberally adminiftered, it may be neceffary to obferve, that the dofes fhould be diminifhed in proportion as the pain abates. Patients of this clafs, as well as thofe afflicted with any other complaint in the urinary paffages, fhould ftudioufly avoid any excefs whatever. A patient feemed to be cured of the ftone by reft, abftemious diet, and mucilaginous medicines; another by the addition of alkaline kaline and acid medicines taken in the act of effervefcence; while foap and liberal dofes of opium have proved of effential fervice, and in others tar water has caufed many calculous fragments to be difcharged, and removed the painful fymptoms.

A fubject proper for lithotomy fhould, except during the fits of pain, enjoy a tolerable ftate of health; be free from conftant fever, or other indifpofition which would render him unfit for another capital operation. In dubious cafes the operation fhould feldom, if ever, be advifed; yet thofe who are the moft improper fubjects are generally the moft urgent to have it recommended: but its importance, and the flight caufes which may produce failure, fhould fufficiently warn us againft giving way to injudicious entreaties. An unpromifing cafe is often pointed out by additional or unufual fymptoms, by continual fever, a white tongue, a fallow countenance, and general weaknefs, together with very turbid, if not dark coloured urine. Under fuch circumftances, the patient fhould be diverted from the operation by its delay, and by a profpect of relief from internal means.

In

In favour of lithotomy it may be remarked, that when due attention has been paid to the neceſſary preceding ſtate of the patient, the diſeaſe ſeldom returns: I know of no inſtance of ſuch a return, except when the ſtone has been unfortunately broken.

When a ſecond operation becomes requiſite, although the lateral method is with propriety generally preferred, yet if no objection appeared to the high operation, I ſhould think it, under ſuch circumſtances, more adviſable.

Infants under two years of age would be too young to undergo an operation of ſuch conſequence as lithotomy; but much greater diſcernment is required in judging of the propriety of recommending it to elderly perſons. I have delayed the operation for ſome months in one patient on account of his youth, and I have operated upon another ſixty-ſeven years of age; but I had reaſon to aſcribe the latter's recovery and ſubſequent enjoyment of health to the ſtrength of his conſtitution.

As it would be highly improper to perform lithotomy during a painful paroxyſm, ſuch means as have been found to alleviate the pain ſhould be for ſome time previouſly continued, and an occaſional aperient ſhould alſo be given.

When the time of operating is fixed upon, and the patient's consent obtained, it is universally agreed that, a few hours before, a clyster should be administered to empty the rectum; and although an experienced lithotomist * has advised the bladder to be also previously emptied, yet other authors † have recommended a distended bladder. When I have directed a patient to empty his bladder immediately before the operation, I have found that urine enough remained to distinguish the proper introduction of the gorget; and when the bladder has been full of urine, either the action of the clyster or the introduction of the staff have made it extremely difficult to pursue their advice. If, however, a quantity of urine can be retained, it may certainly facilitate the operation; although I doubt if the mischievous effects which have been attributed to previous mictition are the result of practice.

As whatever might add to the patient's anxiety should be diligently avoided, the apparatus and office of each assistant should be settled beforehand, and the eyes of the patient

* Mr. Bromfeild.

† Mr. Wilmer, Mr. Bell, &c.

should

ſhould be timely covered. A complete and well-regulated apparatus adds confidence to an operator, proves of eſſential ſervice to a patient, and affords ſatisfaction to all the attendants. In the ſelection of aſſiſtants, no objection ſhould be made to medical judges, as many unforeſeen difficulties may, by ſuch aid, be obviated. Greater deliberation, coolneſs, attention, and reſolution, are requiſite in this than in almoſt any other operation. A perfect acquaintance with all the parts concerned, with the guides for operating, with the knowledge of what parts are to be divided and what avoided, with the poſition and ſecurity of the patient, the mode of operating, and principles of action of the inſtruments employed, as well as the difficulties that have occurred to other lithotomiſts, are circumſtances meriting the greateſt attention. It is fortunate when a young ſurgeon meets with an opportunity of performing this operation, as an elderly ſurgeon, or one who has not ſeen the operation performed, would ſcarcely feel himſelf qualified to commence with ſuch an important undertaking.

If medical ſtudents, or their teachers, verſed in drawing, would accuſtom themſelves to take ſketches of parts concerned in operations from nature, good engravings might be expected, which would prove extremely useful in renewing our anatomical knowledge.

What may be termed guides in the lateral operation are — the raphe, symphysis pubis, tuberosity of the left ischium, anus, and the line in which the staff may be perceived by the touch. The principal parts to be divided are— the integuments on the left side of the raphe, from near the pubis to below the anus; the left accelerator, transversalis, and a portion of the levator ani muscles; the urethra, prostate gland, and vesica urinæ. The parts in danger of being wounded are—the bulb of the urethra, the rectum, pudical artery, and vesicula seminalis. If the parts to be divided could be all distinctly seen, a great part of the difficulty would vanish; but the intimate and accurate knowledge of what is requisite will more evidently appear when it is considered that but a few of the parts are clearly exposed to view during the operation.

Although coolness in a lithotomist may be of great advantage, yet care should be taken to prevent its degenerating into want of feeling, which perhaps may have sometimes happened from his aiming solely at dexterity, and might be avoided by a vigilant attention to every circumſtance that can encourage or be advantageous to the patient, and by guarding againſt any expreſſion that may tend to alarm him. Whilſt want of feeling may produce raſhneſs, humanity is not incompatible with any neceſſary qualification for an operator.

An unremitting attention to every ſtep in the operation will ſupply a lithotomiſt with reſolution, and a great ſhare of it becomes neceſſary from the unavoidable fatigue, eſpecially when any unfavourable circumſtances occur. The points moſt neceſſary to be adverted to during the operation are—the poſition and ſecurity of the patient, and alſo of the ſtaff; the marking, by the eye, the parts recited as guides; the making the external inciſion ſufficiently extenſive; the perfect diviſion of the muſcular parts; the taking care that the ſtaff be denuded, and the beak of the gorget preſerved in the groove of the ſtaff; the proper introduction and withdrawing of the gorget; the paſſing the index finger of the left hand before the introduction of the forceps; the being mindful that the ſtone is ſufficiently within the blades of the forceps, and the poſition of the patient relaxed, before an attempt is made to extract the ſtone; and and laſtly, the purſuing the moſt favourable direction for its extraction.

The modus operandi is accurately deſcribed by different authors *, and ſhould engage the ſtricteſt examination and deliberation before an operation of ſo much importance be undertaken.

Should it be requiſite to reſtrain an hæmorrhage, great advantage will ariſe from the external wound being large. Dry lint ſhould be employed as a dreſſing; and if a bandage is uſed, flannel will be the beſt material. The circumſtance of the bed being of ſuch kind as not to be uncomfortable, or ſpoiled by wetting, ſhould be attended to as well as the poſition of the patient.

Favourable ſymptoms ſucceeding lithotomy are—the abdomen continuing ſoft and eaſy; no great fever or pain; an early and free diſcharge of urine by the wound, the edges of which put on a florid appearance; refreſhing ſleep; and a pleaſure in taking proper nouriſhment. Menacing ſigns are—a pain and tenſion in the region of the bladder; a defect in the urinary diſcharge by the wound, the edges of which become

* Cheſelden, Sharpe, Bromfeild, Bell, Wilmer, &c.

floughy ; an acute fever; lofs of appetite; difturbed fleep, and conftant anxiety.

The fymptomatic fever is generally of an inflammatory kind, and relieved by a ftrict obfervance of antiphlogiftic means; yet in aged perfons they fhould be more cautioufly urged. The warm bath, topical embrocations, fomentations, and bleeding, aperients, antimonials, and anodynes, together with clyfters, and a cooling regimen, are, on fuch occafions, ufually employed.

The urine generally begins to find its natural paffage as foon as the unfavourable fymptoms are removed; but the furgeon's attention fhould not be remitted, not only until the cure is perfected, but for fome little time after, left the imprudence of the patient fhould caufe a lafting inconvenience.

From what has been related it will appear, that great pains muft be taken to acquire fufficient information to qualify a lithotomift; that the operation having been done early, is advantageous; that it fhould not be advifed in dubious cafes; and that a ftrict inquiry fhould be made as to the ftate of the patient and the previous treatment. The many valuable obfervations already publifhed upon this fubject demonftrate monſtrate its importance, and indeed it ſeems almoſt inexhauſtible.

However deplorable the ſituation of thoſe who really labour under this malady may be, yet the caſes, which ſometimes occur, of perſons who erroneouſly imagine themſelves afflicted with it claim our compaſſion. Without any genuine ſigns of ſtone in the bladder, ſuch an obſtinate certainty of its exiſtence is impreſſed upon the mind of ſuch patients, that a compariſon of their complaints with the true ſymptoms, an aſſurance of the difficulty experienced by ſurgeons of forming ſuch an opinion from ſimilar tokens, or ſounding the bladder, ſeldom tends to convince them of their error; and I have even found them come with a decided reſolution to be cut for the ſtone. No adult age ſeems to be exempt from ſuch attacks; nor are they uſually accompanied with any other mental indiſpoſition. In ſome ſuch patients I have no doubt but there is ſome occult complaint in or near the urinary paſſages; and as other diſorders may ſo far reſemble the ſtone as to render the niceſt diſtinction neceſſary, ſome attention ſhould be paid to theſe fictitious ſigns of the diſeaſe, if it be but to prevent the patient's falling falling into the hands of impoftors, and endangering a more hazardous ftate of health.

I have known fuch patients purfue a courfe of lixivia until their conftitutions were irreparably injured. It feems, therefore, incumbent upon the moft fcrupulous practitioners to prefcribe for them, and encourage them with hope of their having an indubitable profpect of cure from early application.

As mixtures containing fixed air have been with good effect tried in real diforders of this kind, and are likely to prove beneficial in ftrengthening the fyftem, they feem proper to be united, in fuch cafes, with uva urfi, bark and other tonics. Occafional anodynes will be found alfo of effential fervice. A more fuccefsful mode of treatment may have been experienced by others. Thofe who have been long in medical practice muft have feen many diforders affect patients in a fimilar manner. The diftinction between vifionary and real fymptoms will feldom be difficult, but the treatment is often fo perplexing, that I have been led to conclude there are few difeafes with which a patient is fancifully afflicted, the real exiftence of which would not be preferable.

These

These hints were originally intended as an answer to a letter from a young surgeon desirous of qualifying himself for the operation of lithotomy; and they are now offered to the Public on a supposition that they may perhaps induce the younger part of the profession to more methodical reflections, and the more experienced to communicate their valuable and instructive remarks on this important part of surgery.

